# Development of an Enzyme-Linked Immunosorbent Assay for Detection of the Native Conformation of Enterovirus A71

**DOI:** 10.1128/msphere.00088-22

**Published:** 2022-06-01

**Authors:** Natalie J. Kingston, Keith Grehan, Joseph S. Snowden, Mona Shegdar, Helen Fox, Andrew J. Macadam, David J. Rowlands, Nicola J. Stonehouse

**Affiliations:** a Astbury Centre for Structural Molecular Biology, School of Molecular and Cellular Biology, Faculty of Biological Sciences, University of Leeds, Leeds, United Kingdom; b Division of Virology, National Institute for Biological Standards and Control, Potters Bar, Hertfordshire, United Kingdom; University of Zurich

**Keywords:** enterovirus A71, sandwich ELISA, scFv, EVA71

## Abstract

Enterovirus A71 (EVA71) is a medically important virus that is commonly associated with hand, foot, and mouth disease (HFMD). It is responsible for periodic outbreaks, resulting in significant economic impact and loss of life. Vaccination offers the potential to control future outbreaks, and vaccine development has been increasingly the focus of global research efforts. However, antigenic characterization of vaccine candidates is challenging because there are few tools to characterize the different antigenic forms of the virus. As with other picornaviruses, EVA71 virions exist in two antigenic states, native (NAg) and expanded (HAg). It is likely that the composition of vaccines, in terms of the proportions of NAg and HAg, will be important for vaccine efficacy and batch-to-batch consistency. This paper describes the development of a single-chain fused variable (scFv) domain fragment and the optimization of a sandwich enzyme-linked immunosorbent assay (ELISA) for the specific detection of the NAg conformation of EVA71. NAg specificity of the scFv was demonstrated using purified EVA71, and conversion of NAg to HAg by heating resulted in a loss of binding. We have thus developed an effective tool for characterization of the specific antigenic state of EVA71.

**IMPORTANCE** EVA71 is a medically important virus that is commonly associated with HFMD, resulting in periodic outbreaks, significant economic impact, and loss of life. Vaccination offers the potential to curtail future outbreaks, and vaccine development has been increasingly the focus of global research efforts. However, antigenic characterization of vaccine candidates is challenging because there are very limited effective tools to characterize the different antigenic forms of EV71. As with other picornaviruses, EVA71 virions exist in two antigenic states, native and expanded. This paper describes the development of an scFv and the optimization of a sandwich ELISA for the specific detection of the native conformation of EVA71 as an effective tool for characterization of the specific antigenic state of EVA71.

## INTRODUCTION

Enterovirus A71 (EVA71) is a medically important member of the family *Picornaviridae*, being one of the main etiological agents of hand, foot, and mouth disease (HFMD). HFMD causes a substantial socioeconomic burden in regions of endemicity and, although this generally causes a mild self-limiting disease in children, outbreaks can be associated with neurological disease and death (1, 2). Indeed, it appears that a combination of host and viral factors is associated with the likelihood of progressing to severe disease. These factors include polymorphisms within host immune and receptor-associated genes (3–5). While there is some cross-reactivity among the different genogroups (A, B1 to B5, and C1 to C5) (6), the antigenic variation makes the synthesis of a pan-EVA71 protective vaccine more difficult.

The EVA71 virion is assembled from 60 copies of three structural proteins, VP0, VP3, and VP1, which form 12 pentameric subunits of an icosahedral shell. A final maturation cleavage of VP0 into VP4 and VP2 occurs during encapsidation of the single-stranded RNA genome. Thus, the presence of VP0 or VP4 and VP2 serves to distinguish between empty capsids (ECs) and mature virions.

The importance of HFMD has encouraged the investigation of a number of approaches to develop vaccines against EVA71, including recombinant VP1 protein, synthetic peptides, virus-like particles (VLPs), live attenuated virus, and inactivated whole virus (7–16), the latter being licensed for use in China. Although such vaccines are effective, the large-scale production of infectious viral material prior to inactivation poses safety concerns; therefore, vaccine manufacturers are looking toward alternative methodologies, including VLPs.

Recombinantly produced VLPs are initially antigenically similar to naturally occurring ECs and are indistinguishable from infectious virus. In the extracellular environment and at moderate temperatures, however, VLPs undergo an antigenic conversion from the native antigen (NAg) conformation to a expanded antigen (HAg) conformation (17). These conformations correspond to the re- and post- receptor engaged states, respectively. For the related and better studied poliovirus (PV), long-term protective responses are generated by NAg particles, while HAg forms do not appear to confer protection (Macadam AJ).

Candidate VLP vaccines for EVA71 have been developed using insect cells (via baculovirus-mediated expression), Pichia pastoris, and Saccharomyces cerevisiae, mainly via the coexpression of the structural precursor polyprotein P1 and the viral protease 3CD. Coexpression of individual structural proteins has also been documented (13–15). When used as immunogens, these VLPs induced protective responses in murine models of EVA71 infection and protected neonatal mice from lethal virus challenge following maternal immunization (13–15). While these VLPs are clearly effective potential vaccines, it remains to be demonstrated whether the NAg and HAg conformations provide different protective efficacies; this question remains unanswered in no small part due to an inability to antigenically characterize the native and expanded antigenic conformations.

The conformational changes associated with particle expansion have been mapped structurally; the HAg form has a greater diameter than the NAg form and has a more angular appearance by negative-stain transmission electron microscopy (TEM) (6, 17, 18). High-resolution structures revealed that antiparallel VP2 helices located on the boundaries between pentamers were separated by approximately 9.3 Å at the 2-fold axes of symmetry in the expanded state, 2.9 Å further apart than in the native conformation (19). In addition, conversion to the expanded form of the particle results in several conformational changes surrounding a cavity in VP1 known as the pocket, with concomitant loss of observable density for the sphingosine-like factor that occupies this site in the NAg state (20). As with other enteroviruses, an extended VP3-GH loop protrudes from the capsid in the expanded conformation. While structurally the conformational changes associated with EVA71 particle expansion are clear, there remains a lack of biochemical assays to determine their antigenic consequences.

A number of enzyme-linked immunosorbent assay (ELISA) methods have been developed for the relative quantification of EVA71 particles, but none of these distinguishes between NAg and HAg forms (14, 21–24). Several factors must be considered for future EVA71 vaccine quality assurance; batch-to-batch consistency, for example, is a critical quality control issue, and precise characterization of vaccine antigens is essential for maintaining consistency. Better characterization of the antigenic state of particles contributing to vaccines, and their capacity to induce neutralizing antibodies, will help us to understand the correlates of protection required for effective EVA71 vaccines.

Here, we present the first description of an anti-EVA71 ELISA capable of distinguishing between native and expanded antigenic conformations, an important characteristic for the development of reliable and consistent EVA71 vaccine batches.

## RESULTS

### Generation of 16-2-2D scFv.

**FIG 1:**
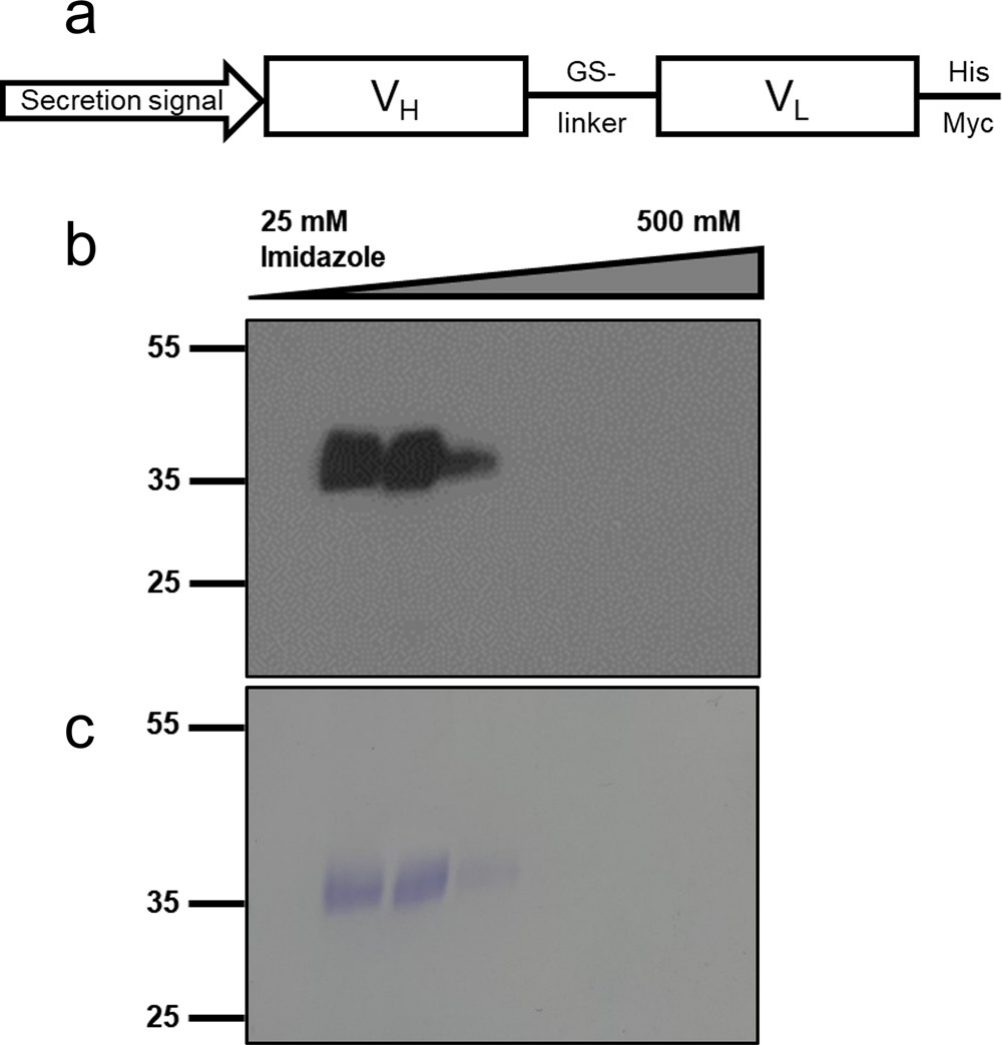
Expression and purification of 16-2-2D scFv. (a) Schematic of the expression cassette used for the production of scFv in P. pastoris. (b and c) Western blotting (b) and Coomassie blue staining (c) to determine the presence of scFv in IMAC elution fractions. Samples were denatured in Laemmli buffer, separated using SDS-PAGE, and visualized by Coomassie blue staining or anti-His Western blotting. Representative data are shown (*n* = 3).

Our preliminary ELISA-based screen using human FAb fragments indicated that clone 16-2-2D was a promising candidate for reactivity with NAg particles. Due to limited reagent availability, single-chain fused variable (scFv) domain fragments were generated from available published EVA71 antibody sequences and expressed in Pichia pastoris (25, 26). To generate the 16-2-2D scFv, we fused the V_H_ domain downstream of the α-factor secretion signal in the pPinkαHC vector. The V_H_ domain was linked to the V_L_ with an 18-amino acid linking sequence, and the sequence was dual-tagged with a 6×His tag and a Myc tag (Fig. 1a). The expression construct was introduced into PichiaPink, and expression and secretion of the scFv were confirmed using Western blotting. The scFv was purified from filtered culture supernatant using immobilized metal affinity chromatography (IMAC). Elution fractions were collected and separated by gel electrophoresis. His-reactive protein was detected by Western blotting (Fig. 1b), and total protein content was visualized by Coomassie blue staining (Fig. 1c). Elution fractions containing scFv underwent buffer exchange, and protein was quantified using bicinchoninic acid (BCA assay).

### Optimization of 16-2-2D scFv ELISA.

**FIG 2:**
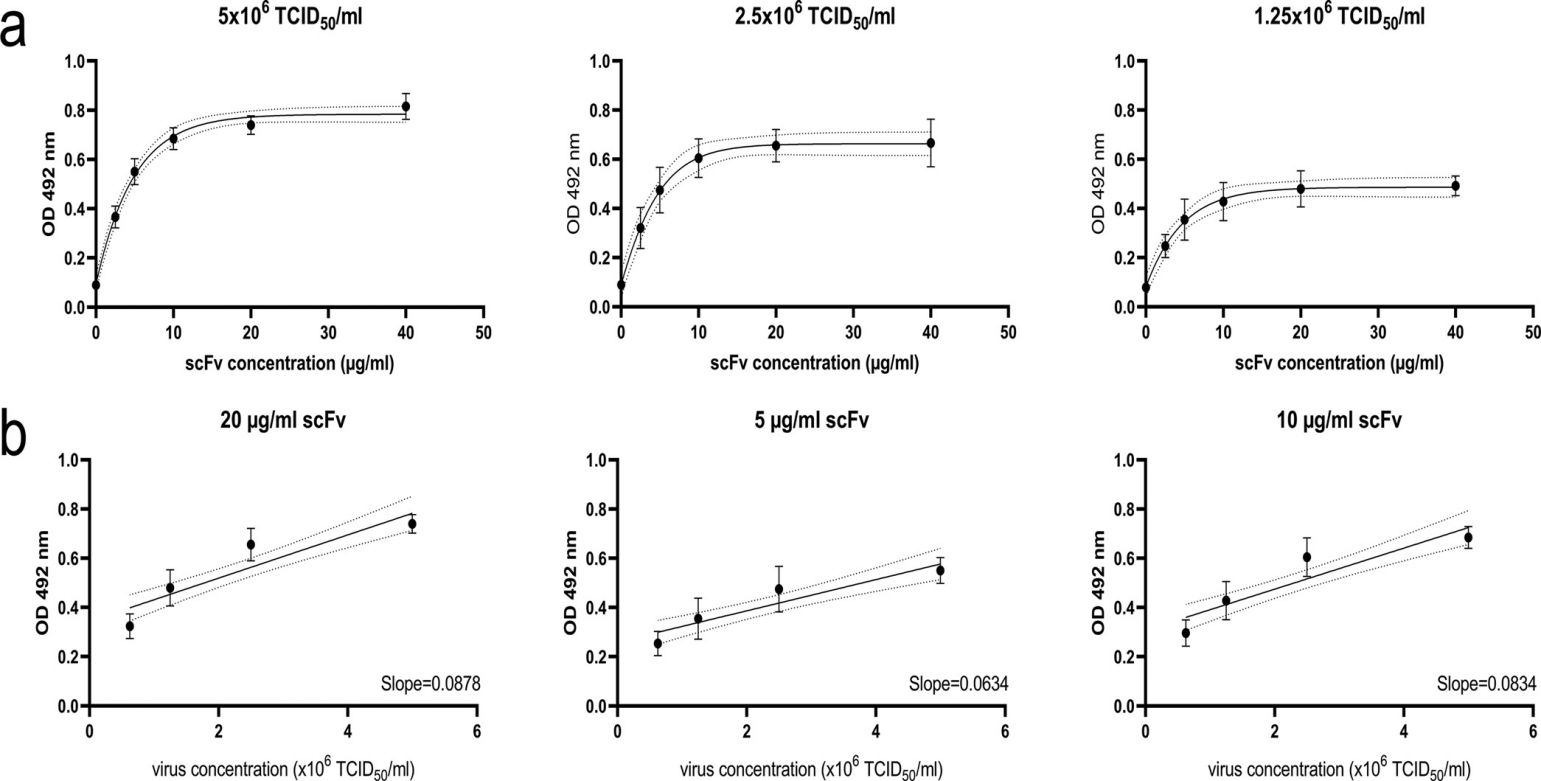
Determining the dynamic range of 16-2-2D scFv. Sandwich ELISA was performed using EVA71 cell culture supernatant and 16-2-2D scFv to identify a suitable working concentration for scFv. (a) Virus concentrations between 5 × 10^6^ and 1.25 × 10^6^ TCID_50_/mL and scFv concentrations between 40 and 2.5 μg/mL were used to identify the linear range of the assay. Graphed data are mean ± standard error of the mean (SEM) (*n* = 3, each in duplicate), and the dotted lines indicate the 95% CI. (b) To determine the suitable working concentration, a linear regression was performed using four successive 2-fold dilutions of EVA71. The linear regression is indicated by the solid line, the dotted lines indicate the 95% CI, and graphed data are mean ± SEM (*n* = 3, each in duplicate).

The ability of recombinantly expressed scFv to recognize EVA71 was confirmed by ELISA, and optimization assays were carried out to determine the dynamic range of the binding interaction. The virus used for these assays was recovered from *in vitro* transcribed RNA from an infectious clone of EVA71, genogroup B2. Recovered virus was passaged in HeLa cells at a multiplicity of infection (MOI) of 0.1, and the titers of clarified supernatant samples were determined by 50% tissue culture infectious dose (TCID_50_) assay before being used for optimization of the ELISA. To determine suitable conditions and identify the dynamic range of the assay, saturating amounts of capture antibody and secondary antibody were used. Doubling dilutions of viral supernatant and scFv were used with virus concentrations ranging from 5 × 10^6^ TCID_50_/mL to 1.25 × 10^6^ TCID_50_/mL and scFv concentrations ranging from 40 μg/mL to 2.5 μg/mL (see Fig. S1 in the supplemental material), and the results of these assays were used to estimate the linear range of the assay (Fig. 2a). At each virus concentration, the near-linear range was between 2.5 μg/mL and 10 μg/mL, and a plateau was apparent between 10 and 40 μg/mL. To verify the optimal scFv concentration, a linear regression was carried out to assess the relationship between the optical density at 492 nm (OD_492_) and the virus concentration. The slope of the regression analysis for 10 μg/mL was 0.0834 (95% confidence interval [CI], 0.06142 to 0.1054) and that for 20 μg/mL was 0.0878 (95% CI, 0.06569 to 0.1099), indicating no significant difference in the capacity of each antibody concentration to detect virus samples (*P* = 0.7706). Using concentrations of 5 μg/mL or less resulted in a significant decrease in the sensitivity of the ELISA (*P* = 0.0049). While the data indicate that all viral concentrations and all scFv concentrations used here were suitable for the detection of virus, we elected to use 10 μg/mL 16-2-2D scFv in all future ELISAs.

10.1128/msphere.00088-22.1 FIG S1 Raw data from scFv ELISA optimization. ELISA to determine the reactivity of the 16-2-2D scFv. scFv concentrations used are along the *x* axis, and virus concentration is indicated in the key; 0 refers to uninfected culture supernatant. Graphed data are mean ± SEM (*n* = 3, in duplicate). Download FIG S1, TIF file, 0.1 MB. Copyright © 2022 Kingston et al. 2022 Kingston et al. https://creativecommons.org/licenses/by/4.0/ This content is distributed under the terms of the Creative Commons Attribution 4.0 International license.

### Specificity of 16-2-2D scFv reactivity with EVA71 virions or ECs.

**FIG 3:**
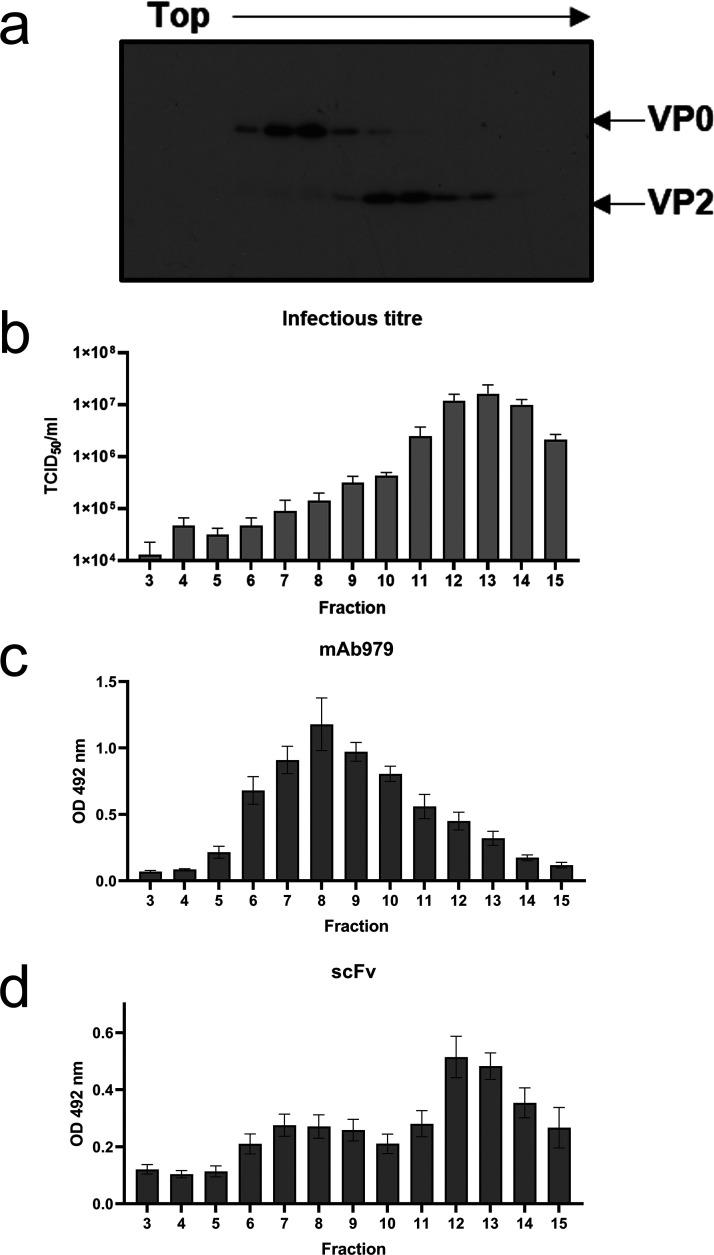
Antigenic specificity of scFv1 and mAb979. (a) Gradient-purified viral samples were assessed for the presence of VP0 and VP2 by Western blotting with MAb 979 (*n* = 3). (b to d) The titers of fractions were determined by TCID_50_ assay (*n* = 3) (b), and sandwich ELISAs were used to determine their reactivity with MAb 979 (c) and scFv (d) (*n* = 3, each in duplicate), with a representative Western blot. Graphed data are mean ± SEM.

Large-scale cultures of EVA71 were purified by sucrose gradient, and fractions were assayed by Western blotting for the presence of VP0 and the cleavage product VP2. ECs (80S), containing VP0, peaked around fraction 8, while the corresponding virions (160S), containing VP2, peaked around fraction 12 (Fig. 3a). Individual fractions were titrated (Fig. 3b) and assessed for the presence of monoclonal antibody (MAb) 979-reactive particles (Fig. 3c) and 16-2-2D scFv-reactive particles (Fig. 3d) by sandwich ELISA. MAb 979 reacts with a linear epitope in the VP2 EF loop that is exposed in the expanded particle conformation, and the reactivity of MAb 979 with ECs is consistent with recognition of this loop being dependent on particle expansion. Conversely, the scFv preferentially bound to fractions containing virus, although some reactivity was seen in fractions primarily associated with ECs, either as a consequence of the incomplete separation of virus and EC, the bispecificity of the scFv, or the presence of both native and expanded conformations of ECs. We carried out similar assays using a genogroup C4 EVA71, but no scFv-specific reactivity was detected against gradient-purified virus or genogroup C4 antigen standard (see Fig. S2). Further assays were performed to better understand the antigenic specificity of the 16-2-2D scFv against genogroup B2 EVA71.

10.1128/msphere.00088-22.2 FIG S2 Genogroup C4 reactivity. Gradient-purified genogroup C4 EVA71 was assessed for reactivity with 16-2-2D scFv (a) and MAb 979 (b) (*n* = 2, in duplicate). Graphed data are mean reactivity with 6.25 × 10^5^ TCID_50_/mL genogroup B2 and 200 AgU/mL The 18/116 international antigen standard (genogroup C4) was assessed against 16-2-2D scFv and MAb CT11F9. Download FIG S2, TIF file, 2.2 MB. Copyright © 2022 Kingston et al. 2022 Kingston et al. https://creativecommons.org/licenses/by/4.0/ This content is distributed under the terms of the Creative Commons Attribution 4.0 International license.

### Determination of the antigenic specificity of 16-2-2D scFv.

To determine whether 16-2-2D is exclusively specific for NAg particles, we carried out antigen conversion assays. The NAg form of enterovirus particles is associated with virus in the infectious conformation; therefore, a loss of infectivity is correlated with a loss of reactivity with an NAg-specific antibody or antibody fragment. Particles undergo antigenic conversion from the NAg form to the HAg form in a number of ways, including receptor engagement, changes in pH or ionic strength, and increases in temperature (17, 19, 20, 27), and we utilized the latter to convert infectious NAg virus to noninfectious HAg virus. This allowed us to determine the specific antigenic reactivity of the 16-2-2D scFv.

**FIG 4:**
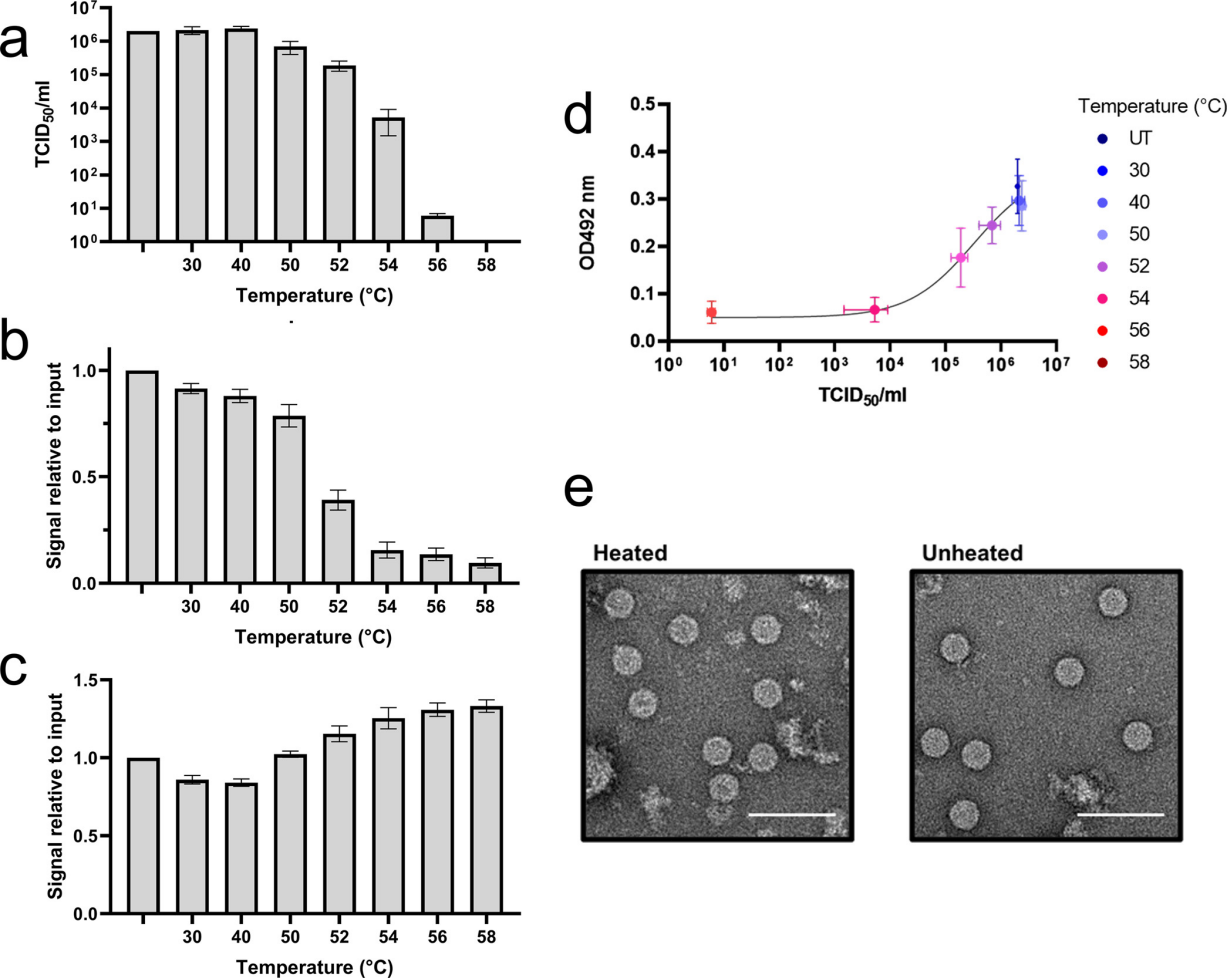
Antigenic conversion of virus. Gradient-purified and concentrated virus samples were heated to a range of temperatures to induce antigenic conversion. (a to c) Graphed infectious viral titers (a), 16-2-2D reactivity (b), and MAb 979 reactivity (c) over a range of incubation temperatures are shown. Data are mean ± SEM (*n* = 3), with ELISAs conducted in duplicate. (d) The relationship between infectious viral titer and 16-2-2D scFv reactivity is graphed. (e) Negative-stain TEM shows particles with or without heating to 55°C. Scale bar = 100 nm.

A loss of infectivity was apparent at temperatures above 50°C, with >90% loss of titer at 52°C and >99.5% loss of titer at 54°C (Fig. 4a). The loss of infectivity was associated with a loss of 16-2-2D scFv-specific reactivity and an increase in MAb 979-specific reactivity (Fig. 4b and c). Indeed, graphing infectious titer versus change in signal supports the relationship between reactivity with the 16-2-2D scFv and infectious titer (Fig. 4d). Importantly, heating of the virus to 55°C does not result in particle disassembly, as shown by negative-stain TEM (Fig. 4e).

## DISCUSSION

While a range of assays are available to assess the yield and purity of EVA71 virus samples, there is a lack of specific assays to define the antigenic state of the particles. Previous ELISAs that are suitable for the detection of EVA71 proteins and particles have been described, although those assays are unable to distinguish the NAg and HAg forms (14, 21–24). In addition, biosafety concerns surrounding the large-scale manufacture of virus for vaccine production are driving the advancement of alternative vaccine approaches, particularly the use of VLPs. Although VLP vaccines have been shown to induce neutralizing and protective antibodies in murine models, the precise antigenic conformation of these particles is not clearly defined (13–15).

As a first step in understanding the importance of different antigenic forms in EVA71 vaccines, we sought to identify an antibody that could bind exclusively to the NAg conformation. To this end, we generated an scFv from the 16-2-2D antibody. The 16-2-2D antibody clone was originally derived from convalescent-phase serum from a genogroup B5-infected individual and was determined to bind to the canyon region of the capsid. It had potent neutralizing ability against the B5 strain but showed limited neutralization of EVA71 genogroup C1 (25, 26, 28). Here, we show that an scFv fragment generated from this sequence can be efficiently produced in P. pastoris (Fig. 1), and we established an ELISA to characterize its binding specificity.

We optimized a sandwich ELISA using 16-2-2D and EVA71 genogroup B2 supernatant (Fig. 2), showing that the scFv reacts with virus-infected supernatant and is nonreactive with noninfected culture supernatant (Fig. 2; also see Fig. S1 in the supplemental material). We also showed that there is no meaningful gain in signal using scFv concentrations of >10 μg/mL, although at lower concentrations a loss of sensitivity is apparent. Cell culture supernatant predominantly contains infectious virus and ECs, but we cannot exclude the possibility that unassembled proteins, pentamers, and assembly or disassembly intermediates are also present in these samples and contribute to the signal detected in the 16-2-2D ELISA. We assessed reactivity with gradient-purified virus to confirm that 16-2-2D reacts with assembled particles (Fig. 3). These data indicated a reactivity profile consistent with the preferential binding of infectious virus, i.e. the NAg conformation (Fig. 3). Conversely, MAb 979 preferentially bound to fractions containing ECs, likely as a consequence of its cognate epitope being exposed in HAg forms but occluded in NAg. When assessed for reactivity against genotype C4 virus, we were unable to detect any 16-2-2D scFv binding (see Fig. S2). This is consistent with the previously described genotype-specific virus neutralization by 16-2-2D (25, 26) and indicates that this ELISA will be unsuitable for genogroup C vaccine characterization. Importantly, however, this ELISA may still allow the fundamental roles of NAg and HAg to be assessed for genogroup B vaccine candidates.

In the better studied PV, the different antigenic forms have functional implications for vaccine efficacy, although it remains unclear whether the same is true for other EVs. While MAb 979 binds to the expanded forms of assembled EVA71 capsids, it still effectively neutralizes virus *in vitro* (12). This is likely a consequence of the substantial overlap of its cognate epitope with the site of SCARB2 binding, thus inhibiting receptor engagement (29).

The functional importance of NAg-specific antibodies against EVA71 remains less clear; although they are not essential for direct neutralization, they may still contribute to the overall immune response, and understanding the role of NAg- and HAg-specific antibodies against EVA71 will help to clarify the precise correlates of protection.

To determine whether 16-2-2D binds NAg preferentially or exclusively, virus samples were heated to convert virus from the NAg form to the HAg form. There was no significant change in infectious titer or reactivity with scFv or MAb 979 when virus samples were heated at 30°C or 40°C. Higher temperatures caused a reduction in infectious titer and a corresponding loss of scFv reactivity along with an increase in MAb 979 reactivity, consistent with the suggestion that 16-2-2D scFv exclusively binds the NAg form (Fig. 4). Negative-stain TEM confirmed the presence of particles in heated samples; therefore, we conclude that the loss of reactivity associated with heating virus samples is a consequence of antigenic conversion and not of particle disassembly. Together, these data indicate that the 16-2-2D scFv is specific for assembled EVA71 particles in the native, infectious conformation and may be an effective and relatively low-cost alternative to MAbs. 16-2-2D antibody fragments will likely be an important tool for characterizing the antigenic state of genogroup B EVA71 and may be useful for future fundamental research and vaccine quality control.

## MATERIALS AND METHODS

### Cells and viruses.

An EVA71 reverse genetics system was generated from genogroup B2 strain MS/7423/87. Viral RNA was extracted from supernatant samples, and the cDNA sequence was reverse transcribed using the Transcriptor first-strand cDNA synthesis kit (Roche, Switzerland). DNA was amplified using sequence-specific primers, and the amplicon was introduced under a T7 promoter and downstream of a hammerhead ribozyme. HeLa cells were obtained from the National Institute for Biological Standards and Control (NIBSC), and Vero cells were obtained from ATCC. PichiaPink strain 1 was purchased from Invitrogen (USA).

### Generating 16-2-2D scFv.

DNA plasmids encoding the heavy and light chain FAb regions of the human antibody 16-2-2D (V_H_, GenBank accession number KY354558.1; V_L_, GenBank accession number KY354572.1) (25) were kindly provided by Daming Zhou and David Stuart (University of Oxford). The scFv sequence was assembled using overlap PCR to generate a single open reading frame encoding the V_H_ domain, a linker sequence, the V_L_ domain, and a dual His/Myc tag. The linker sequence (GGSSRSSSSGGGGSGGGG) was selected to favor the formation of monomeric scFv molecules (30). The scFv was inserted between XhoI and FseI restriction sites within the pPinkαHC vector, utilizing the encoded signal sequence from the vector.

Similar to previously described protocols (31), plasmids were linearized with AflII and electroporated into PichiaPink strain 1 (Invitrogen), and transformed yeast were plated on *Pichia* adenine dropout (PAD) medium and incubated at 28°C for 3 to 5 days. To screen for expression, colonies were selected at random and inoculated into 5 mL YPD medium (10 g/L yeast extract, 5 g/L peptone, 20 g/L dextrose) before incubation at 28°C for 48 h at 250 rpm. Cells were pelleted at 1,500 × *g* and resuspended in 1 mL YPM medium (10 g/L yeast extract, 5 g/L peptone, 2% [vol/vol] methanol). Samples were incubated at 28°C for 72 h at 250 rpm and were supplemented with 2% [vol/vol] methanol every 24 h. Supernatants were collected at 72 h and assessed for scFv production by Western blotting.

For large-scale production, a glycerol stock of 16-2-2D scFv-expressing P. pastoris was used to inoculate 5 mL YPD medium, which was incubated at 28°C for 48 h at 250 rpm. Subsequently, this culture was inoculated into 200 mL of YPD medium and incubated for a further 48 h at 28°C at 250 rpm. Cells were pelleted at 1,500 × *g* and resuspended in 100 mL YPM medium before incubation at 28°C for 72 h at 250 rpm. The medium was supplemented with 2% (vol/vol) methanol every 24 h. Cells were pelleted at 4,000 × *g*, and the supernatant was processed through 0.45-μm and 0.22-μm filters before undergoing His affinity purification.

### Affinity purification.

Filtered scFv samples were processed using IMAC with a 5-mL HisTrap HP His affinity purification column with a flow rate of approximately 1 mL per minute. After the full load volume had passed through the column, 5 mL of 1× phosphate-buffered saline (PBS) was loaded, followed by 5 mL each of 25 mM, 50 mM, 100 mM, 200 mM, 300 mM, 500 mM, and 1,000 mM imidazole in 1× PBS. Wash and elution fractions were collected and assessed for the presence of scFv by Western blotting and Coomassie blue staining.

### Western blot and Coomassie blue staining.

Samples were prepared by mixing 1:1 (vol/vol) sample and 2× Laemmli buffer and denaturing the sample at 95°C for 10 min. Samples were centrifuged at 17,000 × *g* and loaded on 12% (wt/vol) SDS-PAGE gels using standard protocols. To visualize the protein, gels were stained with Coomassie blue (2.5 g/L Coomassie brilliant blue, 90% [vol/vol] methanol, 10% [vol/vol] acetic acid) for 30 min and destained in a 40:10:50 (vol/vol/vol) methanol/acetic acid/water mixture.

Western blot analysis was carried out according to standard protocols; briefly, proteins were transferred to polyvinylidene difluoride (PVDF) membranes and blocked with 5% skim milk powder reconstituted in Tris-buffered saline (TBS) with 0.1% Tween 20. His-reactive proteins were detected using horseradish peroxidase (HRP)-conjugated anti-histidine tag antibody (Bio-Rad, USA) at 1:2,000 dilution. EVA71 VP0/VP2-reactive proteins were detected using anti-EVA71 VP2 antibody clone MAb 979 (Merck, USA) at 1:2,000 dilution and anti-mouse IgG-HRP conjugate. Blots were developed using chemiluminescent substrate (Promega, USA) and X-ray film.

### Virus recovery.

Virus was recovered from *in vitro* transcribed RNA. Briefly, the EVA71 infectious clone plasmids were linearized, followed by phenol-chloroform extraction. RNA was synthesized using the RiboMAX T7 Express large-scale RNA production system (Promega) and purified using RNA Clean and Concentrator columns (Zymo Research, USA). RNA was electroporated into HeLa cells in 0.4-mm cuvettes at 260 V for a single pulse of 25 ms using square wave. Cells were incubated at 37°C in 5% CO_2_ in a humidified chamber overnight. Plates containing cells underwent a freeze-thaw cycle to enhance viral release from cells, cellular debris was pelleted at 17,000 × *g* for 10 min, the viral supernatant was collected, and samples were analyzed using TCID_50_ assays.

### TCID_50_ assays.

To determine the titers of viral samples by TCID_50_ assay, 1 × 10^4^ Vero cells were seeded into each well of a 96-well plate in a volume of 100 μL Dulbecco’s modified Eagle’s medium (DMEM) supplemented with 2% fetal bovine serum (FBS). A series of 10-fold serial dilutions was made from viral supernatants in DMEM supplemented with 2% FBS; 100 μL of each dilution (10^−2^ to 10^−7^) was added to 5 replicate wells. Plates were returned to a 37°C humidified incubator with 5% CO_2_ for 5 days. Samples were fixed and virus was inactivated with the addition of 100 μL 4% formaldehyde for 30 min. The contents of the wells were discarded, and the wells were stained with crystal violet solution. Titers were determined using the Reed-Muench method, and titers are expressed as TCID_50_ per milliliter (32).

### Virus purification.

HeLa cells were seeded in T175 flasks and incubated in DMEM supplemented with 10% FBS until cells reached confluence. Flasks were infected with 2 × 10^6^ TCID_50_ of EVA71 (MOI of ~0.1) and incubated for 48 h when complete cytopathic effect was apparent. Flasks underwent a single freeze-thaw cycle to enhance viral release, samples were centrifuged at 4,000 × *g* for 30 min to pellet cellular debris, and the titers of clarified cell culture supernatants were determined. For smaller-scale genogroup C4 purification, 30 mL of clarified cell culture supernatant was directly pelleted through a 30% sucrose cushion at 150,000 × *g* for 3.5 h. For larger-scale genogroup B2 purifications, 120 mL of viral supernatant (approximately 1.2 × 10^9^ TCID_50_) was precipitated overnight with 8% (wt/vol) polyethylene glycol (PEG) 8000. Precipitated material was pelleted at 4,000 × *g* for 30 min and resuspended in 30 mL PBS. Insoluble material was pelleted at 4,000 × *g* for 30 min, and virus was subsequently pelleted through a 5-mL 30% sucrose cushion at 150,000 × *g* for 3.5 h in a SW32Ti rotor. All virus pellets were resuspended in 1 mL PBS before insoluble material was pelleted at 10,000 × *g* for 10 min and the soluble component was layered atop a discontinuous 15 to 45% sucrose gradient (15, 20, 25, 30, and 45% sucrose in PBS). Gradients were centrifuged at 50,000 × *g* for 12 h, and 1 mL fractions were collected manually from the top of the gradient. The presence of viral proteins was determined by Western blotting and ELISA.

In addition, virus samples for negative-stain TEM and for antigen conversion assays were concentrated and purified. Briefly, samples were added to an Amicon 100,000 molecular weight cutoff (MWCO) polyethersulfone (PES) membrane spin concentrator (Merck) and centrifuged at 2,000 × *g* until the volume had reduced 5-fold (i.e., 100 μL retained). The flowthrough fraction was discarded, and 400 μL PBS was added to the top of the column. This process was repeated four times, and the retained volume was reduced to approximately 30 μL before samples were visualized. Alternatively, samples were collected and diluted in PBS to be used in antigen conversion assays by ELISA.

### Electron microscopy.

To prepare samples for negative-stain TEM, carbon-coated 300-mesh copper grids (Agar Scientific, UK) were glow discharged in air at 10 mA for 30 s. Purified virus (3 μL) was applied to the grids for 30 s, and then excess liquid was removed by blotting. Grids were washed twice with 10 μL distilled water. Grids were stained with 10 μL 2% uranyl acetate solution, which was promptly removed by blotting before another application of 10 μL 2% uranyl acetate solution for 30 s. Grids were subsequently blotted to leave a thin film of stain and then air dried. TEM was performed using an FEI Tecnai F20 transmission electron microscope (operating at 200 kV with a field emission gun) with an FEI Ceta complementary metal oxide semiconductor (CMOS) charge-coupled device camera (Astbury Biostructure Laboratory, University of Leeds). Samples were imaged across a range of defocus values (−1.5 μm to −5 μm) at a nominal magnification of 25,000×, resulting in an object sampling of 0.418 nm/pixel.

### ELISA.

A sandwich ELISA method was utilized to determine the NAg content of viral samples. Briefly, ELISA plates were coated overnight with polyclonal rabbit anti-EVA71 immune sera at a 1:2,000 dilution. Twofold dilutions of clarified virus culture supernatant (5 × 10^6^ TCID_50_/mL to 6.25 × 10^5^ TCID_50_/mL) or PBS alone were added to wells and incubated at 37°C for 1.5 h. Twofold dilutions of scFv (40 μg/mL to 2.5 μg/mL) or PBS alone were added to wells and incubated at 37°C for 1 h, and anti-His-HRP was used to detect scFv at a 1:1,000 dilution (Bio-Rad). Samples were detected using *o*-phenylenediamine dihydrochloride (OPD), and the OD_492_ nm was measured using the Biotek PowerWave XS2 plate reader. Raw data were graphed, and a suitable dynamic range for the scFv was determined to be between 5 μg/mL and 20 μg/mL. All further assays were carried out following the same protocol using an scFv concentration of 10 μg/mL.

To detect expanded antigenic conformations, MAb 979 was used in place of the scFv in the aforementioned method at 1:1,000 dilution (Merck). The presence of MAb 979 was detected using anti-mouse IgG-HRP conjugate as described above.
