# Supplementary figures and images for: Development of an Enzyme-Linked Immunosorbent Assay for Detection of the Native Conformation of Enterovirus A71
Source: mSphere. 2022 Jun 1;7(3):e00088-22. doi: 10.1128/msphere.00088-22 (PMC9241546; doi:10.1128/msphere.00088-22)

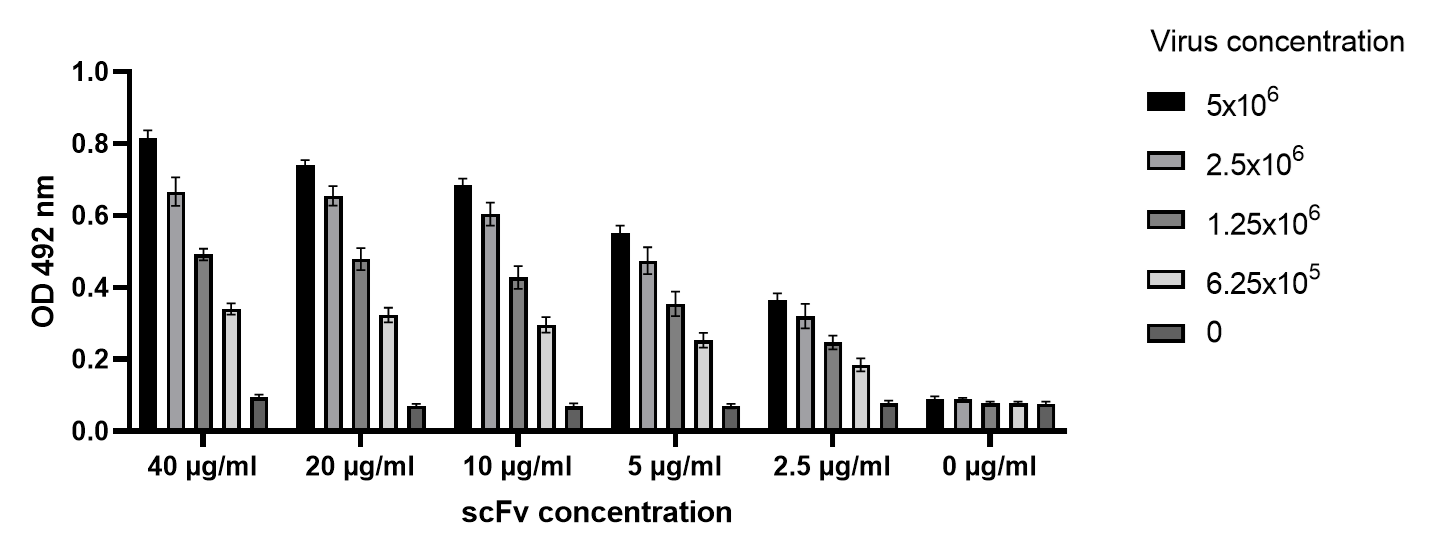

Supplement: FIG S1 [file msphere.00088-22-s0001.tif]

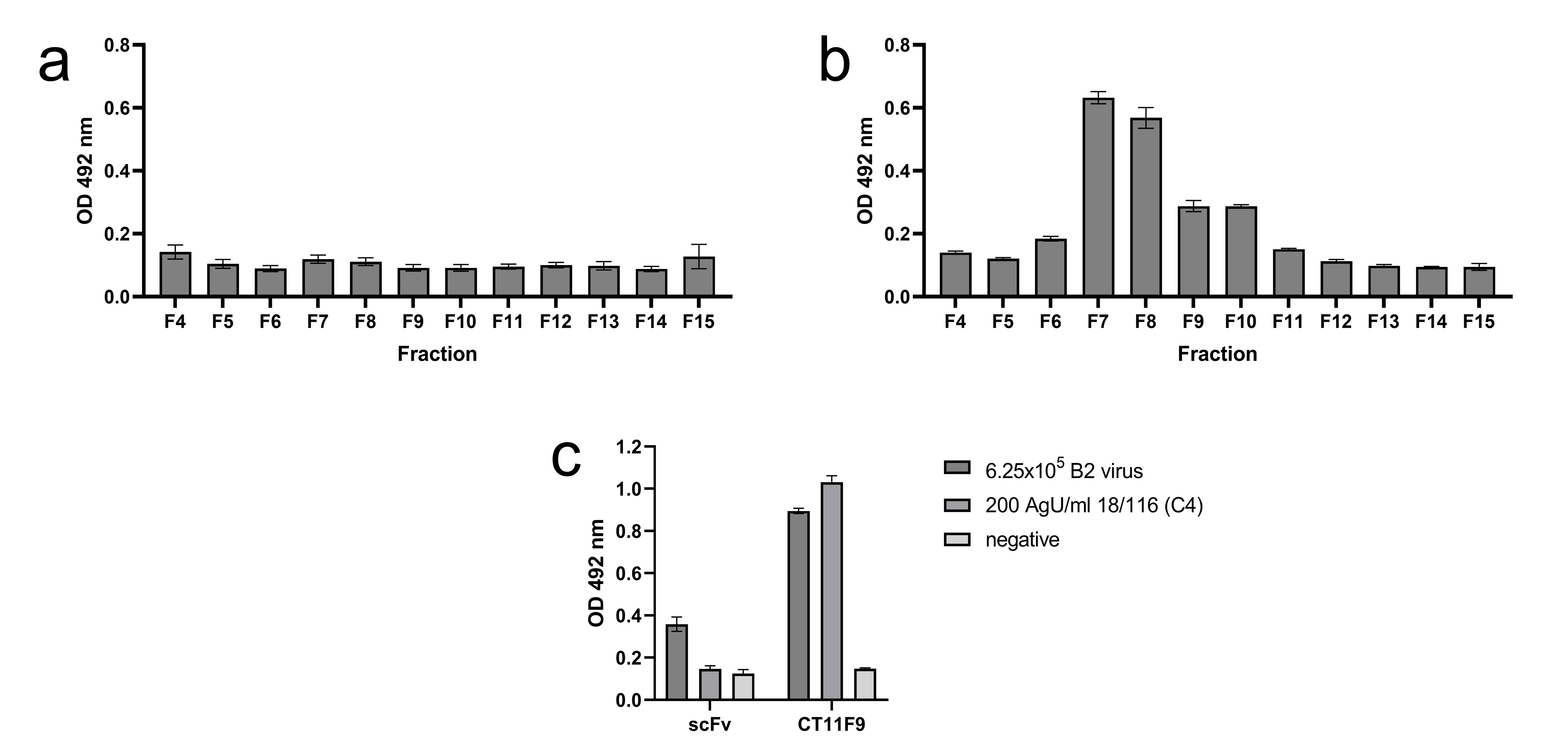

Supplement: FIG S2 [file msphere.00088-22-s0002.tif]
